# Multi-Label Feature Selection Based on High-Order Label Correlation Assumption

**DOI:** 10.3390/e22070797

**Published:** 2020-07-21

**Authors:** Ping Zhang, Wanfu Gao, Juncheng Hu, Yonghao Li

**Affiliations:** 1 College of Computer Science and Technology, Jilin University, Changchun 130012, China; zhangping18@mails.jlu.edu.cn (P.Z.); jchu19@mails.jlu.edu.cn (J.H.); yonghao17@mails.jlu.edu.cn (Y.L.); 2 Key Laboratory of Symbolic Computation and Knowledge Engineering of Ministry of Education, Jilin University, Changchun 130012, China; 3 College of Chemistry, Jilin University, Changchun 130012, China

**Keywords:** multi-label learning, multi-label feature selection, information theory, Max-Correlation

## Abstract

Multi-label data often involve features with high dimensionality and complicated label correlations, resulting in a great challenge for multi-label learning. Feature selection plays an important role in multi-label learning to address multi-label data. Exploring label correlations is crucial for multi-label feature selection. Previous information-theoretical-based methods employ the strategy of cumulative summation approximation to evaluate candidate features, which merely considers low-order label correlations. In fact, there exist high-order label correlations in label set, labels naturally cluster into several groups, similar labels intend to cluster into the same group, different labels belong to different groups. However, the strategy of cumulative summation approximation tends to select the features related to the groups containing more labels while ignoring the classification information of groups containing less labels. Therefore, many features related to similar labels are selected, which leads to poor classification performance. To this end, Max-Correlation term considering high-order label correlations is proposed. Additionally, we combine the Max-Correlation term with feature redundancy term to ensure that selected features are relevant to different label groups. Finally, a new method named Multi-label Feature Selection considering Max-Correlation (MCMFS) is proposed. Experimental results demonstrate the classification superiority of MCMFS in comparison to eight state-of-the-art multi-label feature selection methods.

## 1. Introduction

### 1.1. The Background of Multi-Label Feature Selection

During the past decade, multi-label learning has gradually attracted significant attentions and has been widely utilized in diverse real-world applications, such as text categorization [1,2], information retrieval [3,4] and gene function classification [5,6]. In multi-label data sets, each instance is related to multiple class labels simultaneously. For example, in text categorization tasks, a news document may associate with several topics simultaneously, such as “society”, “economy” and “legality”. Let $X=R^{d}$ denote the *d*-dimensional instance space and $L=\{l_{1},l_{2},..,l_{q}\}$ denote the label space including *q* possible class labels. The task of multi-label learning is to obtain the set of labels related to the unseen instance x∈X by learning a classification model from the training data set $D=\{\left(x_{1},L_{1}\right),\left(x_{2},L_{2}\right),\ldots ,\left(x_{n},L_{n}\right)\}$, where $L_{i}\subseteq L$ is the set of labels associated with $x_{i}$ and $x_{i}\in X\;\left(1\leq i\leq n\right)$ is a *d*-dimensional vector $\left(x_{i1},x_{i2},\ldots ,x_{id}\right)$ [7,8,9]. The classification performance of multi-label learning is closely related to the quality of input features. Like traditional single-label learning algorithm, the multi-label learning often faces with the curse of dimensionality [10].

The high-dimensional multi-label data set often contains a large number of irrelevant and redundant features that bring many disadvantages to the multi-label learning such as the computational burden and over-fitting  [10,11,12]. To address this problem, many multi-label feature selection techniques have been proposed to select the informative feature subset from the original feature set and to discard irrelevant and redundant features [13,14,15]. Feature selection techniques not only reduce the computing costs but also improve the classification performance effectively [16].

Multi-label feature selection methods are usually categorized into three groups: filter methods, wrapper methods and embedded methods [12,17,18,19,20]. Among them, filter methods are classifier-independent, that is, filter methods do not consider any learning algorithm; wrapper methods evaluate the importance of feature subsets based on the classification performance of a specific classifier; embedded methods embed the feature selection in the training process of the classifier. Filter methods have the advantage of low computational cost. In this paper, we focus on filter-based multi-label feature selection methods. In addition, filter methods rank features according to their relevance with the label set. Wrapper methods consider all possible subsets of feature combinations by using the prediction performance of a classifier to assess the quality of feature subsets. Then, the feature subset selected by wrapper methods is optimal for the learning algorithm. The disadvantage of filter methods is that its classification performance is not as good as the wrapper methods, especially in the multi-label feature selection. Therefore, we design a new method to consider the high-order label correlations and to select the most informative features for improving the prediction performance of filter methods.

### 1.2. Information-Theoretical-Based Multi-Label Feature Selection Methods

Different from single-label feature selection methods that evaluate the relevancy between features and only one class label (binary or multiclass), multi-label feature selection methods consider the correlations between features and a set of labels [21,22]. Moreover, the labels in multi-label data are usually not independent, where the internal correlations among labels are always very complicated [23,24]. Many filter-based feature selection methods have been proposed to take into account the label correlations on the evaluation of features, in which information-theoretical-based measures have shown to be adequate [25,26,27,28]. The purpose of the information-theoretical-based multi-label feature selection methods is to obtain an optimal feature subset by employing the information measures in information theory, where mutual information is widely utilized to evaluate the correlation between features and the label set. Suppose that $S=\{f_{1},f_{2},\ldots ,f_{k}\}$ is a feature subset and $L=\{l_{1},l_{2},\ldots ,l_{q}\}$ is the target label set, the mutual information I(S;L) can be denoted as:

(1) $$\begin{array}{cc} I(S;L) & =I(f_{1},f_{2},\ldots ,f_{k};l_{1},l_{2},\ldots ,l_{q})\\ \; & \;=\;\sum_{f_{1}}\;\ldots \;\sum_{f_{k}}\;\sum_{l_{1}}\;\ldots \;\sum_{l_{q}}\;p\left(\;f_{1}\;,\;\ldots \;,\;f_{k}\;,\;l_{1}\;,\;..\;l_{q}\;\right)\;\log\;\frac{\;p(f_{1}\;,\;\ldots \;,\;f_{k},\;l_{1}\;,\;..\;l_{q}\;)\;}{p\left(\;f_{1}\;,\;\ldots \;,\;f_{k}\;\right)p\left(\;l_{1}\;,\;..\;l_{q}\;\right)}. \end{array}$$

The feature subset maximizing Equation (1) provides the maximal information for the label set, which can be considered as the optimal feature subset. However, according to (1), an inevitable problem is that the joint probability p(.) is difficult to estimate accurately. Therefore, many feature selection methods based on low-order label correlations have emerged to obtain the approximate optimal feature subset. Some multi-label feature selection methods  [29,30,31] use the accumulated mutual information between candidate features and each label to evaluate the feature correlations, where these methods consider first-order label correlations, indicating that those labels are independent of each other. Additionally, some methods  [32,33] employ the accumulated conditional mutual information or the interaction information to measure the impact of a candidate feature with each pair of labels, considering second-order label correlations. These methods have been proved to be effective in addressing the curse of dimensionality issues. In fact, there always exist high-order label correlations in the label set that can be abstracted into several semantic groups, in which the same semantic group consists of similar labels and different semantic groups have low dependency. Thus, the cumulative summation approximation based on the whole label set may lead to the following issues:

1. Overestimating the significance of some features when these features have strong correlations with one semantic group containing many labels while being almost independent of the other labels, especially in data with a large collection of labels.
2. Ignoring the key features that are highly correlated with the semantic groups containing less labels.
3. Selecting more redundant features that are often associated with labels in the same semantic group.

In order to address the issues above, we propose a new feature selection method. The main contributions are as follows:

- A new term named Max-Correlation (MC) is designed based on the assumption that labels cluster into several groups, the labels in the same group possess the similar semantic meaning. The MC term employs the maximum operation to select the most informative feature. Additionally, the MC term is not limited to the number of labels in the semantic group, which can effectively address the above issues numbered as 1 and 2.
- We propose a novel feature selection method for multi-label learning based on the Max-Correlation named Multi-label Feature Selection considering the Max-Correlation (MCMFS), which not only maximizes the feature correlation between candidate features and the label set, but also minimizes the feature redundancy in the already-selected feature subset. As a result, our method intends to select the features that are from different semantic groups.
- The effectiveness of the proposed MCMFS method is validated on one artificial data set and twelve real-world multi-label data sets. The experimental results demonstrate that the proposed method can select compact feature subsets and to achieve better classification performance in terms of multiple evaluation criteria.

The remainder of this paper is organized as follows. Section 2 introduces some basic concepts of information theory and four evaluation criteria for multi-label classification performance. Section 3 briefly reviews the related work. In Section 4, we propose the new multi-label feature selection method MCMFS. Section 5 presents the experimental results to verify the effectiveness of the proposed method. In Section 6, we draw conclusions and give the directions of our future research.

## 2. Preliminaries

### 2.1. The Basic Concepts of Information Theory

In this subsection, we introduce some basic concepts of information theory which are used to measure the correlations among random variables [34,35]. Let $X=\{x_{1},x_{2},\ldots ,x_{n}\}$ and $Y=\{y_{1},y_{2},\ldots ,y_{m}\}$ be two discrete random variables. The mutual information measures the amount of information shared by two variables. It is defined as follows:

(2) $$\begin{array}{c} I\left(X;Y\right)\;=\;H\left(X\right)\;-\;H\left(X|Y\right)\;=\;\sum_{i=1}^{n}\;\sum_{j=1}^{m}p\left(x_{i},y_{j}\right)\log\frac{p(x_{i},y_{j})}{p\left(x_{i}\right)p\left(y_{j}\right)}, \end{array}$$

where $p(x_{i},y_{j})$ is the joint probability of $\left(x_{i},y_{j}\right)$, $p(x_{i})$ is the probability of $x_{i}$, $p(y_{j})$ is the probability of $y_{j}$ and the base of log is 2. H(X) is the entropy of the variable *X*, which measures the uncertainty of *X*. H(X|Y) is the conditional entropy of *X* given *Y*, which measures the uncertainty left of *X* under the condition of *Y*. Mutual information can be expressed as the uncertainty reduction about variable *X*, given *Y*. H(X) and H(X|Y) are defined as:

(3) $$\begin{array}{c} H\left(X\right)=-\sum_{i=1}^{n}p\left(x_{i}\right)\log p\left(x_{i}\right), \end{array}$$

(4) $$\begin{array}{c} H\left(X|Y\right)=-\sum_{i=1}^{n}\sum_{j=1}^{m}p\left(x_{i},y_{j}\right)\log p\left(x_{i}|y_{j}\right), \end{array}$$

where $p(x_{i}|y_{j})$ is the conditional probability of $x_{i}$ given $y_{j}$.

Conditional mutual information measures the mutual information between two random variables under the condition of another random variable, which is defined as:

(5) $$\begin{array}{c} I(X;Y|Z)=H(X|Z)-H(X|Y,Z), \end{array}$$

where *Z* is a discrete random variable and H(X|Z) and H(X|Y,Z) are two conditional entropies. The joint mutual information can be defined as:

(6) $$\begin{array}{c} I(X,Y;Z)\;=\;H(Z)\;-\;H(Z|X,Y)\;=\;I(X;Z|Y)\;+\;I(Y;Z). \end{array}$$

Interaction information measures the amount of information shared by three variables, which is defined as:

(7) $$\begin{array}{c} I(X;Y;Z)=I(X;Z)+I(Y;Z)-I(X,Y;Z). \end{array}$$

### 2.2. Multi-Label Evaluation Metrics

To evaluate the classification performance of different multi-label feature selection methods, four evaluation metrics are widely used in multi-label learning in this paper, which are Hamming Loss, Zero-One Loss, Macro-average and Micro-average [36].

Let $D=\{\left(x_{1},L_{1}\right),\left(x_{2},L_{2}\right),\ldots ,\left(x_{n},L_{n}\right)\}$ be a multi-label test set and $L=\{l_{1},l_{2},\ldots ,l_{q}\}$ be the label set, where *n* is the number of instances and $L_{i}\subseteq L$ is the label set corresponding to the instance $x_{i}$. Suppose that $L_{i}^{\prime }$ is the predicted label set corresponding to the $x_{i}$ instance obtained by multi-label classifier.

Hamming Loss (HL) calculates the average fraction of misclassified labels. HL is defined as:

(8) $$\begin{array}{c} HL=\frac{1}{n}\sum_{i=1}^{n}\frac{|L_{i}^{\prime }⊕L_{i}|}{q}, \end{array}$$

where ⊕ denotes the symmetric difference between the label sets $L_{i}$ and $L_{i}^{\prime }$. For example, let $L=\{l_{1},l_{2},l_{3},l_{4},l_{5}\}$. Suppose that $L_{i}=\left\{l_{1},l_{3},l_{5}\right\}$ and $L_{i}^{\prime }=\left\{l_{1},l_{2},l_{5}\right\}$. $L_{i}$ corresponds to vector v=(1,0,1,0,1) where $v_{j}=1$ or 0 (j=1,2,..,5) means that $l_{j}$ is included or not included in $L_{i}$. $L_{i}^{\prime }$ corresponds to vector $v^{\prime }=\left(1,1,0,0,1\right)$. Then, $|L_{i}^{\prime }⊕L_{i}\left|=\right|v^{\prime }⊕v|=2$, where ⊕ is true if ${v^{\prime }}_{j}\neq v_{j}$.

Zero-One Loss (ZOL) calculates the average fraction of instances whose most confident label is not in the relevant label set. The definition for ZOL is:

(9) $$\begin{array}{c} ZOL=\frac{1}{n}\sum_{i=1}^{n}\delta \left(argmax_{l\in L}h\left(x_{i},l\right)\right), \end{array}$$

where δ=1 if $argmax_{l\in L}h\left(x_{i},l\right)\notin L_{i}$ and 0 otherwise. $h(x_{i},l)$ is the real-valued function based on the multi-label classifier, which returns the confidence of label *l* being proper label of $x_{i}$. $argmax_{l\in L}h\left(x_{i},l\right)$ corresponds to the most confident label for $x_{i}$.

Macro-average (Macro-F1) and Micro-average (Micro-F1) based on the F1 score are two widely adopted evaluation criteria for multi-label learning. Macro-F1 is an arithmetic average of the F1 score of all *q* labels. Macro-F1 can be obtained as follows:

(10) $$\begin{array}{c} Macro-F1=\frac{1}{q}\sum_{i=1}^{q}\frac{2TP_{i}}{2TP_{i}+FP_{i}+FN_{i}}, \end{array}$$

where $TP_{i}$, $FP_{i}$ and $FN_{i}$ denote the number of true positives, false positives and false negatives in the *i*-th label, respectively. Micro-F1 can be considered as a weighted average of the F1 over all *q* labels:

(11) $$\begin{array}{c} Micro-F1=\frac{\sum_{i=1}^{q}2TP_{i}}{\sum_{i=1}^{q}\left(2TP_{i}+FP_{i}+FN_{i}\right)}. \end{array}$$

The multi-label classification performance can be measured using the mentioned above evaluation criteria. For the four evaluation criteria, a lower value of HL and ZOL indicates a better classification performance. On the other hand, the higher the Macro-F1 and Micro-F1 values are, the better the classification performance is.

## 3. Related Work

Conventional multi-label feature selection methods can be divided into two groups to deal with the multi-label data sets: problem transformation and algorithm adaptation [37,38]. The problem transformation methods include two steps: (1) transform the multi-label data set to numerous single-label data sets; (2) select the relevant features from the transformed data sets. Binary Relevance (BR) [39], Label Power set (LP) [40] and Pruned Problem Transformation (PPT) [41] are common problem transformation methods. BR decomposes the multi-label data set into several independent binary classification data sets. LP assigns each instance’s label set to a single new class. N. Spolaôr et al. [42] propose four multi-label feature selection methods based on BR and LP which employ ReliefF (RF) [43] and Information Gain (IG) [44] as the feature evaluation criteria to measure the transformed data (RF-BR, RF-LP, IG-BR and IG-LP). However, BR ignores the label correlations and LP may create too many classes causing over-fitting and imbalance problems. PPT removes the instances with rarely occurring labels by a predefined minimal number of occurrences of the label set to improve the effectiveness of LP. Doquire and Verleysen [45] propose a multi-label feature selection method based on mutual information using PPT (PPT + MI). In addition, $\chi^{2}$ statistics are used to select the effective features (PPT + CHI) [41]. However, the problem transformation-based multi-label feature selection methods usually ignore the correlations among labels or lose the label information.

In recent years, many algorithm adaptation-based multi-label feature selection methods that directly select features from the multi-label data set have been proposed. S Kashef and H Nezamabadi-pour [15] propose a multi-label feature selection algorithm based on the Pareto dominance concept that intends to select the label-specific features in multi-objective optimization problem. Sun et al. [26] propose a novel Mutual-Information-based feature selection method via constrained Convex Optimization (MICO), which obtains the discriminative features considering the label correlation. Multi-label Informed Feature Selection (MIFS) [46] is an embedded-based feature selection method that decomposes the multi-label information into a low-dimensional label space using Latent Semantic Indexing (LSI) and then employs the reduced label space to steer the feature selection process via a regression model. Lee and Kim  [32] propose a multi-label feature selection method based on information theory named Pairwise Multi-label Utility (PMU). Its evaluation function is defined as follows:

(12) $$\begin{array}{c} J\left(\;f_{k}\;\right)\;=\;\;\sum_{l_{i}\in L}\;I\;\left(f_{k};l_{i}\;\right)\;-\;\;\sum_{f_{j}\in S}\;\sum_{l_{i}\in L}\;I\;\left(\;f_{k};f_{j};l_{i}\;\right)\;-\;\;\sum_{l_{i}\in L}\;\sum_{l_{j}\in L}\;I\;\left(\;f_{k};l_{i};l_{j}\;\right), \end{array}$$

where $f_{k}$ is a candidate feature, *S* is an already-selected feature subset and $f_{j}$ is a member of *S*, $l_{i}$ and $l_{j}$ are two members of the label set *L*. The PMU method selects the feature $f_{k}$ with the largest value of $J(f_{k})$. Multi-label feature selection method using interaction information (D2F) [29] is proposed to measure the feature correlation between features and each label in the label set. The criterion of D2F is defined as follows:

(13) $$\begin{array}{c} J\left(f_{k}\right)=\sum_{l_{i}\in L}I\left(f_{k};l_{i}\right)-\sum_{f_{j}\in S}\sum_{l_{i}\in L}I\left(f_{k};f_{j};l_{i}\right). \end{array}$$

In addition, Scalable Criterion for a Large Label Set (SCLS) [30] is proposed to design a new multi-label feature selection method based on scalable relevance evaluation. It is denoted as follows:

(14) $$\begin{array}{c} J\left(f_{k}\right)=\sum_{l_{i}\in L}I\left(f_{k};l_{i}\right)-\sum_{f_{j}\in S}\frac{I(f_{k};f_{j})}{H(f_{k})}\sum_{l_{i}\in L}I\left(f_{k};l_{i}\right). \end{array}$$

Lin et al. [31] propose a multi-label feature selection method based on Max-Dependency and Min-Redundancy (MDMR) that maximizes the feature dependency between candidate features and each label using mutual information and minimizes the feature redundancy between candidate feature and each already-selected feature. The criterion of MDMR is denoted as follows:

(15) $$\begin{array}{c} J\;\left(f_{k}\;\right)\;=\;\sum_{l_{i}\in L}\;I\left(f_{k};l_{i}\right)\;-\;\frac{1}{\left|S\right|}\;\sum_{f_{j}\in S}\;\{\;\left(\;I\left(f_{k};f_{j}\right)\;-\;\sum_{l_{i}\in L}\;I\left(f_{k};l_{i}|f_{j}\right)\;\right\}, \end{array}$$

where |S| is the number of features in the already-selected feature subset S. In addition, multi-label Feature Selection based on Label Redundancy (LRFS) [33] is proposed, and LRFS employs the conditional mutual information between candidate features and each label given other labels to consider the measurement of feature relevancy. It is defined as follows:

(16) $$\begin{array}{c} J\left(f_{k}\right)=\sum_{l_{i}\in L}\left\{\sum_{l_{i}\neq l_{j},l_{j}\in L}I\left(f_{k};l_{j}|l_{i}\right)-\frac{1}{\left|S\right|}\sum_{f_{j}\in S}I\left(f_{k};f_{j}\right)\right\}. \end{array}$$

Through the above introduction, we can find that previous information-theoretical-based multi-label feature selection methods employ the cumulative summation approximation to take first-order and second-order label correlations into account. In fact, there exist high-order label correlations in the real-world multi-label data sets, naturally, labels cluster into several groups. The common limitation of these methods is that the cumulative summation may overestimate the significance of some candidate features that are related to the groups containing more labels while ignoring the classification information of groups containing few labels. To explore and exploit accurately high-order correlations among labels, we first design a Max-Correlation (MC) term based on the assumption that similar labels cluster into the same groups and dissimilar labels belong to different groups. Then, we propose a novel method named Multi-label Feature Selection considering the Max-Correlation (MCMFS).

## 4. MCMFS: Multi-Label Feature Selection Considering the Max-Correlation

### 4.1. Proposed Method

Many information-theoretical-based multi-label feature selection methods apply various low-order approximations to evaluate the candidate features. D2F, SCLS and MDMR methods [29,30,31] employ the accumulated mutual information to quantify the contribution of features to the label set. The specific equation is as follows:

(17) $$\begin{array}{c} Rel=\sum_{l_{i}\in L}I\left(f_{k};l_{i}\right). \end{array}$$

**Figure 1:**
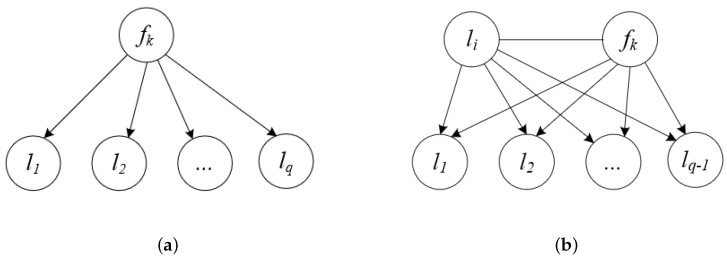
The correlation between feature $f_{k}$ and the label set for the first-order and second-order label correlations.

Equation (17) assumes that labels are independent of each other in the design of the feature relevancy term, which can be described as shown in Figure 1a, where $f_{k}$ is a candidate feature and $l_{i}\in L\;\left(i=1,2,\ldots ,q\right)$ is one label. In addition, conditional mutual information and interaction information are also used to consider the impact of candidate features with each pair of labels $\left(l_{i},l_{j}\right)$, such as PMU [32] and LRFS [33], which can be described as shown in Figure 1b.

(18) $$\begin{array}{c} Rel=\sum_{l_{i}\in L}\sum_{l_{j}\in L}I\left(f_{k};l_{i};l_{j}\right), \end{array}$$

(19) $$\begin{array}{c} Rel=\sum_{l_{i}\in L}\sum_{l_{i}\neq l_{j},l_{j}\in L}I\left(f_{k};l_{j}|l_{i}\right). \end{array}$$

Figure 1 displays the first-order correlations and the second-order correlations among labels. However, label correlations are complicated and of high-order nature in real-world data sets. The labels can naturally cluster into several abstracted semantic meanings. For example, in text categorization, the topics “Athletics”, “Gymnastics” and “Swimming” can be extracted as the semantic meaning “Sports”, and the topics “Beach”, “Sea” and “Mountain” can be extracted as the semantic meaning “Nature”. The labels in the same semantic group have larger dependency, while labels in different semantic meanings are more distinctive. In the literature [25], the 45 labels in the medical data set, which has been used in Computational Medicine Centers 2007 Medical Natural Language Processing Challenge, are divided into 4 main groups according to the statistical information about the labels. Different groups are almost independent of each other and the number of labels in different groups is not equal. Actually, we expect to select the features that are highly discriminating to each semantic group, thereby obtaining the representative features for different semantic meaning.

Like the Equations (17)–(19), the cumulative summation of information terms tends to select features that are related to one semantic group, which leads to overestimating some feature significance especially when the number of labels in the same semantic group is large. As a result, many redundant features are selected. For example, suppose that the total number of labels is 100 and there are two main semantic groups in the label set, that are $C_{1}$ and $C_{2}$. If the number of labels in $C_{1}$ is 90 and the number of labels in $C_{2}$ is 10, then the cumulative summation criterion prefers to select the features that are associated with the labels in the semantic group $C_{1}$, while reducing the selection possibility of features that are from $C_{2}$. In such a situation, the critical features are neglected that are highly related to the semantic groups containing few labels because the value of the cumulative summation will be small when these features are independent of most other labels. Additionally, the selection possibility of redundant features will increase due to the overestimation of the features significance when these features are associated with the same semantic group that contains many labels. However, an effective and compact feature subset should choose features that are from different semantic groups, which is proved to be effective [47].

**Figure 2:**
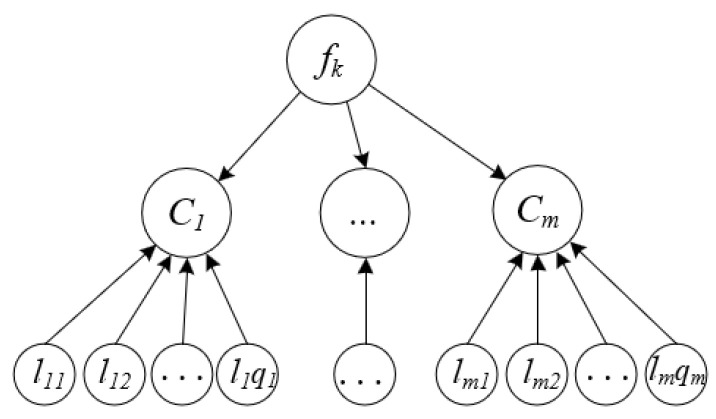
The correlation between feature $f_{k}$ and the label set for the high-order label correlations.

To address the issue, we propose a new multi-label feature selection method to select features that are from different semantic groups. Suppose that the label set $L=\{l_{1},l_{2},\ldots ,l_{q}\}$ can be divided into *m* semantic groups, that is, $L^{\prime }=\left\{C_{1},C_{2},\ldots ,C_{m}\right\}$, where each semantic group $C_{i}=\left\{l_{i1},l_{i2},\ldots ,l_{iqi}\right\}\subseteq L\;\left(i=1,2,\ldots ,m\right)$ and it satisfies $C_{1}\cup C_{2}\cup \ldots \cup C_{m}=L$ and $C_{i}\cap C_{j}=⌀$. Our aim is to select the critical features that are from each semantic group, which is described as shown in Figure 2.

In order to avoid the overestimation problem caused by the number of labels in the different semantic groups, we employ the maximum operation to measure the mutual information between the candidate feature $f_{k}$ and each semantic group $C_{i}\;\left(i=1,2,..,m\right)$. The specific equation is as follows:

(20) $$\begin{array}{c} R\left(f_{k},C_{i}\right)=\max_{l_{j}\in C_{i}}\left(I\left(f_{k};l_{j}\right)\right). \end{array}$$

Equation (20) measures the relevancy between the candidate feature and labels in the same semantic group. The larger the value of Equation (20), the more important the candidate feature is in the semantic group. Equation (20) is the upper bound of the maximal relevancy between one candidate feature and the labels in the semantic group. Furthermore, a small value of Equation (20) means that the relevancy between the candidate feature and the labels in the semantic group is weak. Finally, Equation (20) can effectively avoid the overestimation caused by accumulation, even if many labels are in the same semantic group.

Thereafter, according to Equation (20), an *m*-dimensional vector $\mathrm{Cor}(f_{k};L^{\prime })$ of feature $f_{k}$ and the label set $L^{\prime }$ is obtained, that is $\mathrm{Cor}(f_{k};L^{\prime })=\left[R\left(f_{k},C_{1}\right),R\left(f_{k},C_{2}\right),\ldots ,R\left(f_{k},C_{m}\right)\right]$. We select the maximum value of $\mathrm{Cor}(f_{k};L^{\prime })$ as the feature relevancy between candidate features and the entire label set *L*, which is named Max-Correlation (MC). It is defined as:

(21) $$\begin{array}{cc} MC(f_{k};L) & =\max_{C_{i}\in L^{\prime }}R\left(f_{k},C_{i}\right)\\ \; & =\max_{C_{i}\in L^{’}}\left\{\max_{l_{j}\in C_{i}}I\left(f_{k};l_{j}\right)\right\}=\max_{l_{j}\in L}I\left(f_{k};l_{j}\right). \end{array}$$

$MC(f_{k};L)$ can effectively capture the maximum amount of contribution of the feature regarding the label set. Meanwhile, $MC(f_{k};L)$ can accurately select the critical features whatever how many labels in the semantic groups. Based on the definition of Max-Correlation, we propose a novel multi-label feature selection method named Multi-label Feature Selection considering the Max-Correlation (MCMFS). The evaluation function is as follows:

(22) $$\begin{array}{cc} J(f_{k})\; & =MC\left(f_{k};L\right)\;-\;\frac{1}{\left|S\right|}\sum_{f_{j}\in S}I\left(f_{k};f_{j}\right)\\ \; & =\;\max_{l_{j}\in L}I\left(f_{k};l_{j}\right)-\frac{1}{\left|S\right|}\sum_{f_{j}\in S}I\left(f_{k};f_{j}\right). \end{array}$$

$I(f_{k};f_{j})$ measures the feature redundancy between the candidate feature $f_{k}$ and each already-selected feature $f_{j}$. $\frac{1}{\left|S\right|}$ is employed to balance the magnitude between the Max-Correlation term and the feature redundancy term. Therefore, Equation (22) uses $MC(f_{k};L)$ to maximize the feature relevancy between candidate features and the label set, while using the mutual information $I(f_{k};f_{j})$ to minimize the feature redundancy in the already-selected feature subset to choose the feature that are from different semantic groups. The sequential forward search strategy is used in the process of feature selection. We select the feature $f_{k}$ that achieves the maximum value of $J(f_{k})$ as the next already-selected feature. The pseudo code of MCMFS is presented in Algorithm 1.
**Algorithm 1** MCMFS**Input:**  A training sample *D* with a full feature set $F=\{f_{1},f_{2},\ldots ,f_{d}\}$ and the label set $L=\{l_{1},l_{2},\ldots ,l_{q}\}$;   The number of selected features *b*. **Output:**
  The already-selected feature subset *S*. 1:S←⌀; 2:a←0; 3:**for** i=1 to *d* **do** 4: According to the Equation (21) calculate the $MC(f_{i};L)$; 5:**end for** 6:**while** a<b **do** 7: **if** *a* == 0 ** then** 8:  Select the feature $f_{j}$ with the largest $MC(f_{i};Y)$; 9:  a=a+1;10:  $S=S\cup \{f_{j}\}$;11:  $F=F-\{f_{j}\}$;12: **end if**13: **for** each candidate feature $f_{i}\in F$ **do**14:  Calculate the mutual information $I(f_{i};f_{j})$;15:  According to the Equation (22) calculate the $J(f_{i})$;16: **end for**17: Select the feature $f_{j}$ with the largest $J(f_{i})$;18: $S=S\cup \{f_{j}\}$;19: $F=F-\{f_{j}\}$;20: a=a+1;21:**end while**


There are three stages in the MCMFS method. In the first stage (lines 1–5), it initializes the parameters, which includes the already-selected feature subset *S* and the number of already-selected features *a* in lines 1–2, and calculates the Max-Correlation for each feature in lines 3–5. The second stage (lines 7–12) selects the maximum value of Max-Correlation as the first already-selected feature. The third stage (lines 13–20) calculates the Equation (22) and selects the next feature.

The minimal-redundancy-maximum-relevance (mRMR) [48] is a well-known single-label feature selection method, which uses mutual information between candidate features and class labels to evaluate feature relevance and adopts the same feature redundancy term with our method. The resemblance between mRMR method and MCMCFS method is that both methods consider the relationship between candidate features and already-selected features to minimize feature redundancy. The difference is that mRMR method does not consider the effects of label correlations. In multi-label feature selection, the proposed MCMCFS method employs the Max-Correlation term to consider the high-order label correlations.

### 4.2. Complexity Analysis

We provide the complexity analysis for the MCMFS method and other five information-theoretical-based feature selection methods (D2F, PMU, SCLS, MDMR and LRFS). Suppose that the number of instances is *n*, the number of features is *d* and the number of labels is *q*. The mutual information, conditional mutual information and interaction information need the time complexity of O(n) since all the instances need to be visited for probability estimation. Suppose that the number of selected features is *b*, then the time complexity of MCMFS and SCLS is O(ndq+bnd). The time complexity of D2F and MDMR is O(ndq+bndq). PMU and LRFS design the evaluation criteria to consider the second-order label correlations. The time complexity of PMU is $O(ndq+bndq+ndq^{2})$ and the time complexity of LRFS is $O(ndq^{2}+bnd)$. Table 1 lists the time complexity of these methods. As shown in Table 1, MCMFS achieves the same time complexity with SCLS method. In addition, the time complexity of MCMFS method is lower than that of the D2F, MDMR, PMU and LRFS methods. Therefore, the proposed method is more computationally efficient than these four methods.

## 5. Experimental Results and Analysis

**Figure 3:**
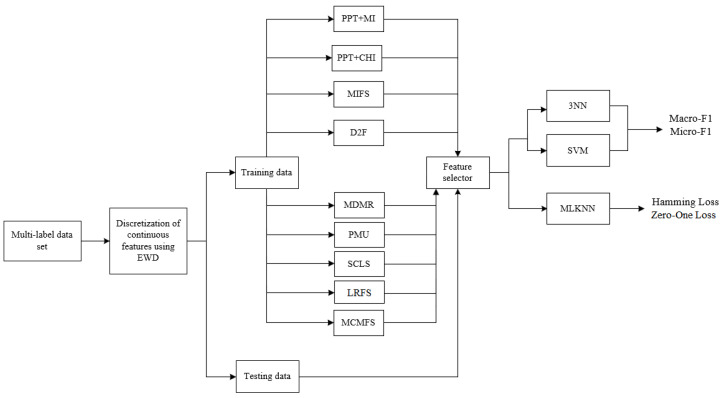
Experimental framework.

In this section, we evaluate the classification performance of the proposed MCMFS method and present the experimental results. MCMFS is compared to one embedded-based method (MIFS [46]) and two problem transformation-based methods (PPT + MI [45] and PPT + CHI [41]) and five information-theoretical-based methods (D2F [29], MDMR [31], PMU [32], SCLS [30] and LRFS [33]). First, we introduce the experimental settings and describe the evaluation framework in Figure 3. Second, MCMFS is compared to five information-theoretical-based methods that employ the cumulative summation approximation to evaluate the candidate features on an artificial data set. Finally, the MCMFS method is compared to the eight representative methods on 12 real-word multi-label data sets in terms of four evaluation metrics to verify the effectiveness of MCMFS method. All the experiments are executed on an Intel Core (TM) i7-6700 with 3.4 GHz processing speed.

### 5.1. Experimental Setting

The experimental setting is as follows: First, the continuous features are discretized using an equal-width strategy into three bins, as recommend in the literature [14,29]. Second, the number of already-selected features *b* varies from 1 to M with a step size of 1, where M is 20% of the total number of features (M = 17% in medical data set used in experiments). Third, we employ the MLKNN [47] as the multi-label classifier to evaluate the classification performance of the MCMFS method and other eight compared feature selection methods in terms of Hamming Loss and Zero-One Loss. Additionally, the number of nearest neighbors *K* is set to 10. Finally, k-Nearest Neighbors (kNN) and Liblinear-based Support Vector Machine (SVM) are implemented to evaluate the classification performance in terms of Macro-F1 and Micro-F1. The kNN is a non-linear neighborhood-based classifier, while the SVM is a linear classifier. We adopt two different classifiers to display the different classification performance of these methods. In addition, kNN and SVM are two popular classifiers in the feature selection methods based on information theory, they are widely applied in various literature [49,50,51,52,53]. Different k values of kNN classifier appear to have less effect on the classification performance for the filter methods [53]. In these references, *k* is set to 3, indicating that this is an empirical setting. Therefore, we set *k* to 3 in this paper. We use the package scikit-learn and in Python 2.7 to implement the classifiers. The multi-label data sets used in the experiment are from Mulan Library [46] where the training set and test set have been already separated in the data source. Therefore, as shown in Figure 3, we use the result of feature selection on the training set to implement on test set directly.

### 5.2. Experiment and Analysis on an Artificial Data Set

We apply an artificial data to visually compare MCMFS to five information-theoretical-based methods (D2F, LRFS, MDMR, PMU and SCLS) that employ the cumulative summation approximation to evaluate the importance of candidate features. Table 2 displays the artificial data D={O,F,L}, where $O=\{o_{1},o_{2},\ldots ,o_{10}\}$, $F=\{f_{1},f_{2},\ldots ,f_{8}\}$ and $L=\{l_{1},l_{2},\ldots ,l_{5}\}$.

The matrix of mutual information between labels D(L,L) is listed. It can be observed that $l_{1}$, $l_{2}$ and $l_{3}$ have close correlations, and they have weak correlation with $l_{4}$ and $l_{5}$. In addition, $l_{4}$ and $l_{5}$ are also weakly correlated with each other. For example, for the label $l_{1}$, the values of $I(l_{1};l_{2})$ and $I(l_{1};l_{3})$ are significantly greater than the values of $I(l_{1};l_{4})$ and $I(l_{1};l_{5})$. For the label $l_{4}$, all the values of $I(l_{4};l_{1})$, $I(l_{4};l_{2})$, $I(l_{4};l_{3})$ and $I(l_{4};l_{5})$ are very small. Intuitively, the label set can cluster into three semantic groups, that are $C_{1}=\left\{l_{1},l_{2},l_{3}\right\}$, $C_{2}=\left\{l_{4}\right\}$ and $C_{3}=\left\{l_{5}\right\}$.

(23) $$\begin{array}{cc} D(L,L) & =\left[\begin{array}{ccccc} I(l_{1};l_{1}) & I(l_{1};l_{2}) & I(l_{1};l_{3}) & I(l_{1};l_{4}) & I(l_{1};l_{5})\\ I(l_{2};l_{1}) & I(l_{2};l_{2}) & I(l_{2};l_{3}) & I(l_{2};l_{4}) & I(l_{2};l_{5})\\ I(l_{3};l_{1}) & I(l_{3};l_{2}) & I(l_{3};l_{3}) & I(l_{3};l_{4}) & I(l_{3};l_{5})\\ I(l_{4};l_{1}) & I(l_{4};l_{2}) & I(l_{4};l_{3}) & I(l_{4};l_{4}) & I(l_{4};l_{5})\\ I(l_{5};l_{1}) & I(l_{5};l_{2}) & I(l_{5};l_{3}) & I(l_{5};l_{4}) & I(l_{5};l_{5}) \end{array}\right]\\ \; & =\left[\begin{array}{ccccc} 0.971 & 0.256 & 0.256 & 0.006 & 0.006\\ 0.256 & 0.971 & 0.256 & 0.006 & 0.006\\ 0.256 & 0.256 & 0.971 & 0.006 & 0.006\\ 0.006 & 0.006 & 0.006 & 0.881 & 0.002\\ 0.006 & 0.006 & 0.006 & 0.002 & 0.881 \end{array}\right] \end{array}$$

We present the feature ranking results and the classification performance obtained by the six feature selection methods in Table 3. Five-fold cross-validation is employed to evaluate the classification performance on the artificial data. The values in bold font represent the best classification performance in Table 3. It can be seen that MCMFS obtains better experimental results in terms of HL, ZOL, Macro-F1 and Micro-F1. According to the results of feature ranking, the five compared methods give lesser importance rankings for certain features. For example, compared to D2F, LRFS, MDMR and SCLS, the rank of $f_{8}$ is higher in MCMFS. In fact, $f_{8}$ is the most relevant feature to the label $l_{5}$ ($f_{8}=argmax_{f_{i}\in F}\left(I\left(f_{i};l_{5}\right)\right)$). Compared to D2F, LRFS and PMU, the rank of $f_{3}$ is higher in MCMFS, where $f_{3}$ is the most relevant feature to the label $l_{4}$ ($f_{3}=argmax_{f_{i}\in F}\left(I\left(f_{i};l_{4}\right)\right)$). In other words, $f_{8}$ and $f_{3}$ are critical features of the semantic groups $C_{3}$ and $C_{2}$, respectively. $f_{2}$ is the key feature of the semantic group $C_{1}$ that is selected by most methods. The proposed method finds accurately the key features that belong to different semantic groups.

### 5.3. Experimental Results on the Real-Word Data Sets

The experiments are conducted on 12 real-world multi-label data sets that are from Mulan Library [54]. The description of the data sets is presented in Table 4. These data sets contain different number of instances, features and labels. In addition, these data sets cover two different application areas. The data set scene is collected for semantic image categorization and the remaining data sets are widely applied to text categorization.

Table 5 and Table 6 record the average classification results and standard deviations of the proposed method and other eight compared methods on the 12 data sets in terms of Hamming Loss and Zero-One Loss, respectively. The values in bold font represent the best classification performance achieved by the corresponding method.

In Table 5, MCMFS obtains the best Hamming Loss performance on 11 data sets. MIFS method provides better results for the Business data set, which means that the decomposition process of the label set of the MIFS method is helpful for the feature selection on the Business data set. As shown in Table 6, PPT + CHI obtains better performance than the proposed MCMFS method and other compared methods on Reference data set in terms of Zero-One Loss performance. MCMFS obtains the best Zero-One Loss performance on 11 data sets. On the whole, MCMFS provides better classification performance compared to other competitive feature selection methods in terms of Hamming Loss and Zero-One Loss on MLKNN classifier.

Table 7, Table 8, Table 9 and Table 10 record the classification performance of the proposed method and other eight compared methods in terms of Macro-F1 and Micro-F1, respectively. Table 7 and Table 8 present the Macro-F1 metric on the SVM classifier and 3NN classifier, respectively. As the results indicate, we can observe that D2F obtains the best Macro-F1 performance on the enron data set using the SVM classifier in Table 7. Our method outperforms the compared methods in terms of the Macro-F1 performance on 11 data sets using the SVM classifier and on 12 data sets using the 3NN classifier. Table 9 and Table 10 show the Micro-F1 performance on the SVM classifier and 3NN classifier, respectively. Compared to the eight methods, MCMFS obtains the best Micro-F1 performance on 11 data sets using the SVM classifier. In Table 10, MCMFS obtains the best Micro-F1 performance on 9 data sets using the 3NN classifier. Overall, our method achieves the best classification performance in terms of Macro-F1 and Micro-F1 on these data sets using the SVM classifier and 3NN classifier.

Observing these results, PPT+CHI provides better classification performance on Reference data set in terms of ZOL, Macro-F1 on SVM classifier and 3NN classifier, Micro-F1 on SVM classifier. $\chi^{2}$ is effective in evaluating the features of Reference data set by transforming the label set to single label using PPT. Compared with the information-theoretical-based methods, the classification performance of MCMFS is is the best among all methods, followed by LRFS, MDMR, D2F, PMU and SCLS, which verifies the effectiveness of using the maximum operation instead of the cumulative summation approximation to take into account the higher-order label relationship.

**Figure 4:**
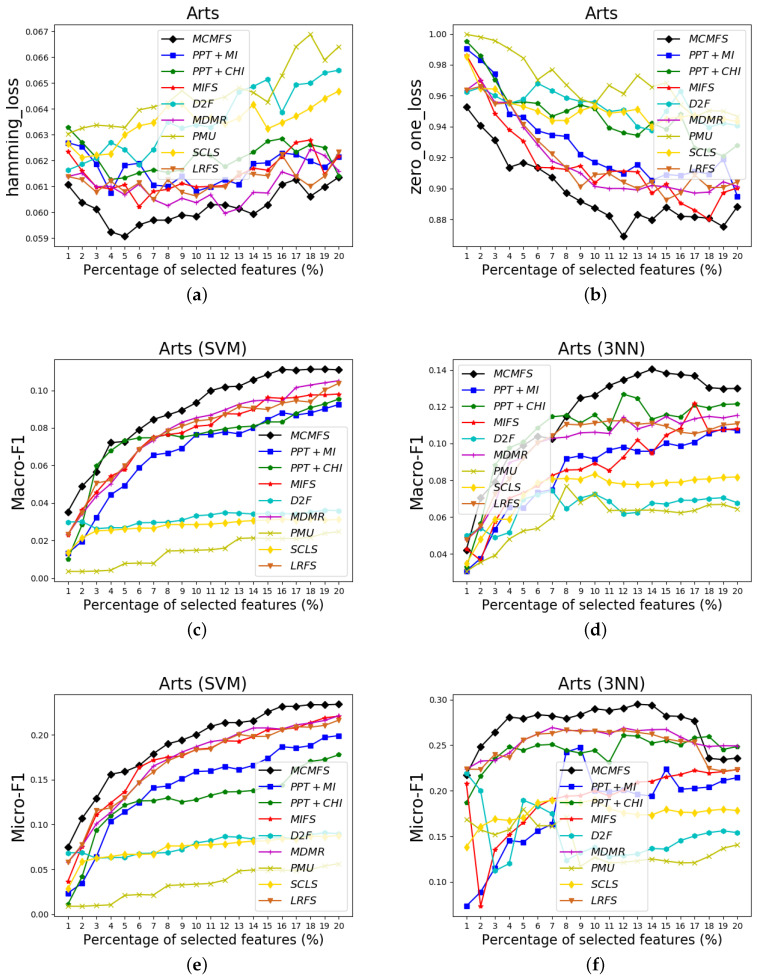
Classification performance on Arts data set: (**a**) Hamming Loss, (**b**) Zero-One Loss, (**c**) Macro-F1 on SVM classifier, (**d**) Macro-F1 on 3NN classifier, (**e**) Micro-F1 on SVM classifier, (**f**) Micro-F1 on 3NN classifier.

**Figure 5:**
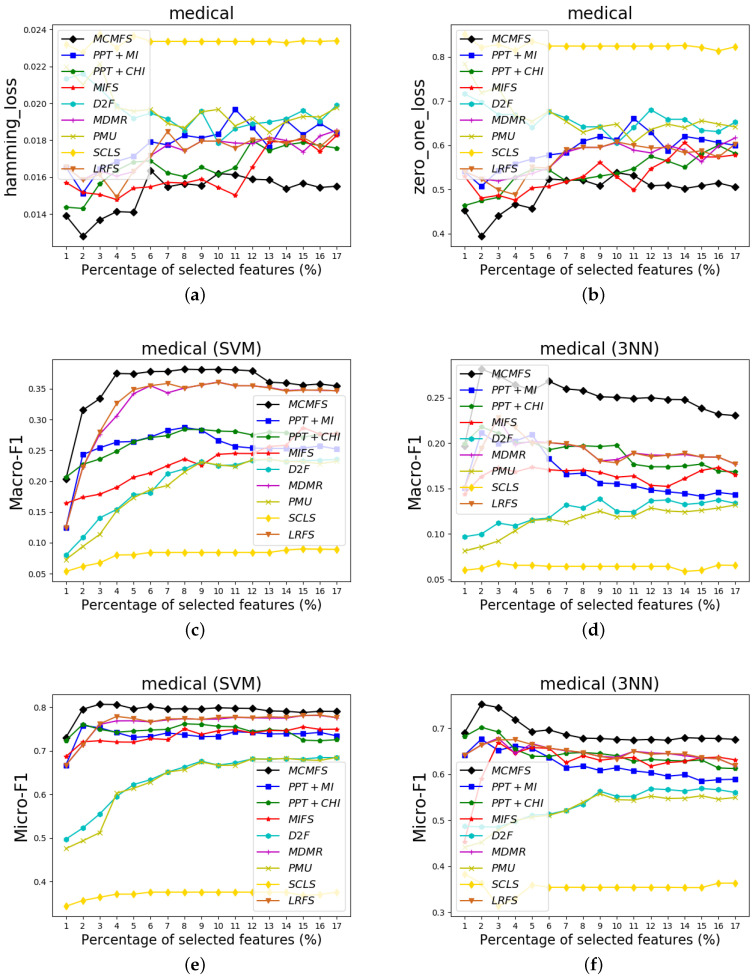
Classification performance on medical data set: (**a**) Hamming Loss, (**b**) Zero-One Loss, (**c**) Macro-F1 on SVM classifier, (**d**) Macro-F1 on 3NN classifier, (**e**) Micro-F1 on SVM classifier, (**f**) Micro-F1 on 3NN classifier.

**Figure 6:**
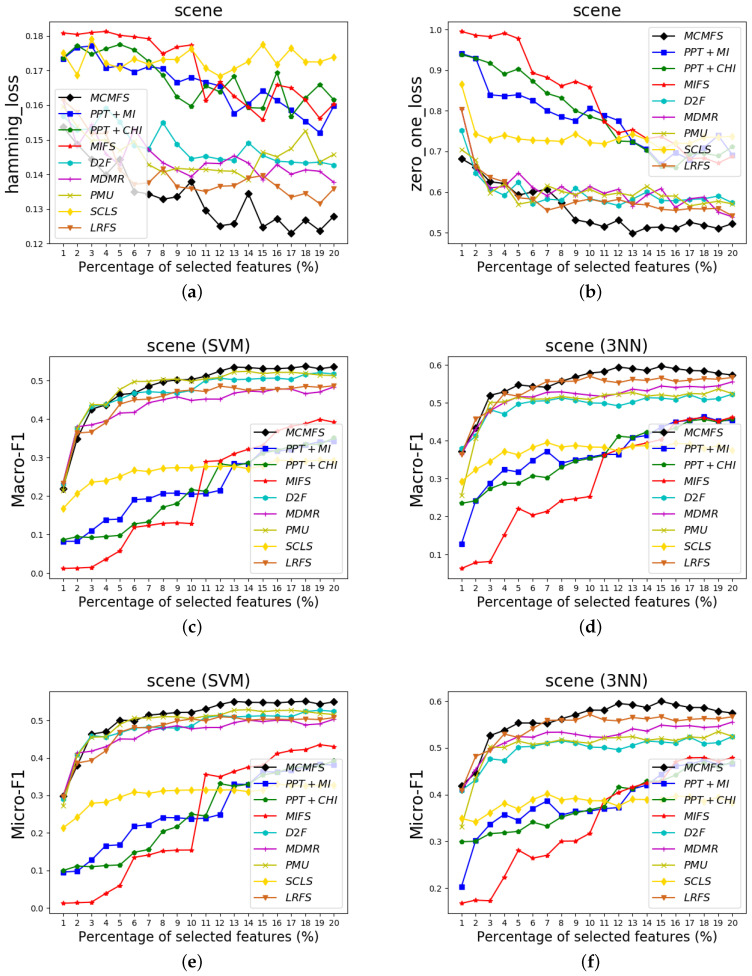
Classification performance on scene data set: (**a**) Hamming Loss, (**b**) Zero-One Loss, (**c**) Macro-F1 on SVM classifier, (**d**) Macro-F1 on 3NN classifier, (**e**) Micro-F1 on SVM classifier, (**f**) Micro-F1 on 3NN classifier.

To clearly show the classification performances of different feature selection methods, Figure 4, Figure 5 and Figure 6 show the experimental results on three data sets (Arts, medical and scene). In these figures, the *X*-axis represents the number of already-selected features, which is varied as {1%, 2%, …, 20%} or {1%, 2%, …, 17%} (medical data set) of the total number of features. The *Y*-axis represents the experimental results of the different evaluation criteria. Different colors and shapes indicate different multi-label feature selection methods.

According to the classification performance on the Figure 4, Figure 5 and Figure 6, we can observe that MCMFS obtains better classification performance than other compared feature selection methods. Compared to five information-theoretical-based methods D2F, MDMR, PMU, SCLS and LRFS, the experimental results demonstrate that the maximum operation is more effective than the cumulative summation approximation operation. In addition, MCMFS outperforms the other three multi-label feature selection methods PPT + MI, PPT + CHI and MIFS on these data sets.

**Figure 7:**
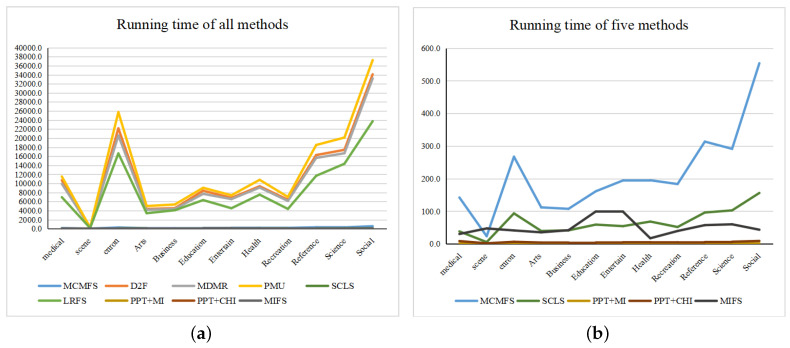
The running time of nine methods: (**a**) all methods, (**b**) Multi-label Feature Selection considering the Max-Correlation (MCMFS), Pruned Problem Transformation (PPT) + mutual information (MI), PPT + CHI, Multi-label Informed Feature Selection (MIFS) and Scalable Criterion for a Large Label Set (SCLS).

Finally, we show the running time of MCMFS and other eight compared methods in Table 11. The running time of PPT + MI and PPT + CHI methods is the minimum, because they only need one iteration on the transformed single label to complete the feature selection. Although SCLS and MIFS methods have lower running time than our method, the proposed method outperforms these two methods in terms of multiple evaluation criteria for the classification performance. As compared to D2F, MDMR, PMU and LRFS, our method is more computationally efficient. Therefore, the running time of MCMFS method is generally acceptable. Additionally, we use Figure 7 to present the minimum and maximum values of each method on different data sets. In Figure 7, the *X*-axis represents data sets while the *Y*-axis represents the running time of each method. To clearly show the running time of different methods, we use Figure 7b to display the running time of MCMFS, PPT + MI, PPT + CHI, MIFS and SCLS methods. As shown in Figure 7, we can find that PMU obtains the most running time among all methods. PPT + MI has the least running time. The running time of our method MCMFS is acceptable. In addition, the running time of all methods increases as the size of the data sets increases.

## 6. Conclusions

In this paper, a novel multi-label feature selection method is proposed named Multi-label Feature Selection considering the Max-Correlation (MCMFS). The Max-Correlation (MC) term is designed based on the high-order label correlations and the assumption that labels naturally cluster into several groups. The combination of maximum operation and the feature redundancy term contributes to selecting the features that are from different label groups.

To demonstrate the effectiveness of our method, MCMFS is compared to five information-theoretical-based multi-label feature selection methods (D2F, MDMR, PMU, SCLS and LRFS) that employ the cumulative summation approximation operation to select features on an artificial data set. Furthermore, MCMFS is compared to eight state-of-the art multi-label feature selection methods (PPT + MI, PPT + CHI, MIFS, D2F, MDMR, PMU, SCLS and LRFS) using MLKNN on 12 real-world multi-label data sets in terms of Hamming Loss and Zero One Loss. Additionally, the 3NN classifier and SVM classifier are used to evaluate the classification performance among the nine feature selection methods in terms of Macro-F1 and Micro-F1. The experimental results demonstrate that MCMFS obtains better classification results than the compared methods and can effectively select a compact feature subset for the classification.

Finally, in our future work, we intend to explore high-order label correlations and sparse learning for multi-label feature selection. Additionally, we intend to propose a method that can automatically assign the appropriate number of feature subsets to each data set.

## Figures and Tables

**Table 1:** The time complexity of six methods.

Methods	Time Complexity
MCMFS	O(ndq+bnd)
SCLS	O(ndq+bnd)
D2F	O(ndq+bndq)
MDMR	O(ndq+bndq)
PMU	$O(ndq+bndq+ndq^{2})$
LRFS	$O(ndq^{2}+bnd)$

**Table 2:** An artificial data.

O	$f_{1}$	$f_{2}$	$f_{3}$	$f_{4}$	$f_{5}$	$f_{6}$	$f_{7}$	$f_{8}$	$l_{1}$	$l_{2}$	$l_{3}$	$l_{4}$	$l_{5}$
$o_{1}$	0	1	0	0	0	1	0	0	1	1	1	0	0
$o_{2}$	1	0	0	0	0	1	1	1	0	0	0	0	1
$o_{3}$	1	0	1	1	0	1	1	0	0	0	0	1	0
$o_{4}$	1	1	0	0	0	0	1	1	1	1	1	1	1
$o_{5}$	0	0	1	0	1	0	1	0	0	0	0	1	0
$o_{6}$	0	1	0	0	0	0	0	0	1	1	1	0	0
$o_{7}$	0	0	0	0	0	0	1	0	0	0	0	0	1
$o_{8}$	0	0	0	1	0	0	0	0	1	0	0	0	0
$o_{9}$	1	0	0	0	0	1	0	0	0	1	0	0	0
$o_{10}$	0	0	0	0	1	0	1	0	0	0	1	0	0

**Table 3:** Experimental results on the artificial data set.

Methods	Feature Ranking	HL↓	ZOL↓	Macro-F1 (SVM) ↑	Micro-F1 (SVM) ↑	Macro-F1 (3NN) ↑	Micro-F1 (3NN) ↑
MCMFS	$\left\{f_{2},f_{8},f_{3},f_{7},f_{4},f_{5},f_{6},f_{1}\right\}$	**0.3075**	**0.8875**	**0.4017**	**0.4651**	**0.3783**	**0.4694**
D2F	$\left\{f_{2},f_{7},f_{1},f_{6},f_{8},f_{4},f_{3},f_{5}\right\}$	0.3433	0.9583	0.3342	0.3985	0.2350	0.3148
LRFS	$\left\{f_{2},f_{7},f_{8},f_{3},f_{1},f_{4},f_{5},f_{6}\right\}$	0.3625	0.9292	0.3550	0.4191	0.2883	0.3829
MDMR	$\left\{f_{2},f_{7},f_{3},f_{8},f_{4},f_{5},f_{1},f_{6}\right\}$	0.3342	0.9333	0.3533	0.4261	0.3617	0.4646
PMU	$\left\{f_{8},f_{7},f_{1},f_{6},f_{2},f_{4},f_{5},f_{3}\right\}$	0.3758	0.9875	0.2733	0.3395	0.1917	0.2823
SCLS	$\left\{f_{2},f_{7},f_{3},f_{4},f_{1},f_{6},f_{8},f_{5}\right\}$	0.3408	0.9583	0.3517	0.4321	0.3375	0.4220

**Table 4:** Description of data sets.

No.	Data Set	#Instances	#Features	#Labels	#Training	#Test
1	medical	978	1449	45	333	645
2	scene	2407	294	6	1211	1196
3	Enron	1702	1001	53	1123	579
4	Arts	5000	462	26	2000	3000
5	Business	5000	438	30	2000	3000
6	Education	5000	550	33	2000	3000
7	Entertain	5000	640	21	2000	3000
8	Health	5000	612	32	2000	3000
9	Recreation	5000	606	22	2000	3000
10	Reference	5000	793	33	2000	3000
11	Science	5000	743	40	2000	3000
12	Social	5000	1047	39	2000	3000

**Table 5:** Experimental results of multi-label feature selection methods in terms of Hamming Loss (HL) (mean ± std).

Data set	MCMFS	PPT + MI	PPT + CHI	MIFS	D2F	MDMR	PMU	SCLS	LRFS
medical	**0.015 ± 0.001**	0.018 ± 0.001	0.017 ± 0.002	0.017 ± 0.002	0.02 ± 0.001	0.018 ± 0.001	0.02 ± 0.001	0.023 ± 0	0.018 ± 0.001
scene	**0.135 ± 0.011**	0.167 ± 0.006	0.167 ± 0.007	0.17 ± 0.01	0.149 ± 0.006	0.145 ± 0.007	0.147 ± 0.007	0.173 ± 0.003	0.142 ± 0.01
enron	**0.051 ± 0.002**	0.053 ± 0.002	0.059 ± 0.001	0.057 ± 0.001	0.052 ± 0.001	0.053 ± 0.003	0.052 ± 0.001	0.053 ± 0.001	0.055 ± 0.003
Arts	**0.060 ± 0.001**	0.062 ± 0.001	0.062 ± 0.001	0.061 ± 0.001	0.064 ± 0.001	0.061 ± 0.001	0.064 ± 0.001	0.063 ± 0.001	0.061 ± 0.001
Business	0.029 ± 0.000	0.029 ± 0.001	0.029 ± 0.000	**0.028 ± 0.000**	0.029 ± 0.001	0.029 ± 0.001	0.029 ± 0.000	0.029 ± 0	0.029 ± 0.001
Education	**0.042 ± 0.001**	0.043 ± 0.001	0.043 ± 0.001	0.044 ± 0.001	0.044 ± 0.001	0.043 ± 0.001	0.045 ± 0.001	0.044 ± 0.001	0.043 ± 0.001
Entertain	**0.061 ± 0.001**	0.064 ± 0.001	0.065 ± 0.001	0.066 ± 0.001	0.066 ± 0.001	0.063 ± 0.002	0.067 ± 0.001	0.066 ± 0.001	0.063 ± 0.001
Health	**0.044 ± 0.001**	0.046 ± 0.001	0.045 ± 0.002	0.05 ± 0.001	0.048 ± 0.001	0.045 ± 0.001	0.049 ± 0.001	0.049 ± 0.001	0.045 ± 0.001
Recreation	**0.061 ± 0.001**	0.062 ± 0.001	0.062 ± 0.001	0.062 ± 0.001	0.062 ± 0.001	0.062 ± 0.001	0.065 ± 0.001	0.064 ± 0.001	0.061 ± 0.001
Reference	**0.031 ± 0.001**	0.032 ± 0.001	0.032 ± 0.001	0.031 ± 0.001	0.032 ± 0.001	0.031 ± 0.001	0.034 ± 0.001	0.033 ± 0	0.031 ± 0.001
Science	**0.035 ± 0.001**	0.036 ± 0.001	0.036 ± 0.000	0.036 ± 0.000	0.036 ± 0.000	0.035 ± 0.000	0.036 ± 0.000	0.036 ± 0.000	0.035 ± 0.001
Social	**0.026 ± 0.001**	0.028 ± 0.001	0.03 ± 0.001	0.032 ± 0.001	0.03 ± 0.001	0.028 ± 0.001	0.031 ± 0	0.029 ± 0.001	0.027 ± 0.001
Average	**0.049**	0.053	0.054	0.055	0.053	0.051	0.053	0.055	0.051

**Table 6:** Experimental results of multi-label feature selection methods in terms of Zero-One Loss (ZOL) (mean ± std).

Data set	MCMFS	PPT + MI	PPT + CHI	MIFS	D2F	MDMR	PMU	SCLS	LRFS
medical	**0.50 ± 0.05**	0.59 ± 0.05	0.55 ± 0.06	0.55 ± 0.08	0.66 ± 0.04	0.58 ± 0.04	0.66 ± 0.04	0.83 ± 0.01	0.58 ± 0.04
scene	**0.57 ± 0.08**	0.78 ± 0.08	0.8 ± 0.09	0.83 ± 0.12	0.61 ± 0.06	0.61 ± 0.07	0.61 ± 0.07	0.74 ± 0.04	0.6 ± 0.08
enron	**0.89 ± 0.02**	0.9 ± 0.03	0.98 ± 0	0.98 ± 0.01	0.9 ± 0.02	0.91 ± 0.03	0.9 ± 0.03	0.94 ± 0.03	0.93 ± 0.04
Arts	**0.90 ± 0.03**	0.93 ± 0.03	0.95 ± 0.02	0.92 ± 0.03	0.95 ± 0.01	0.92 ± 0.03	0.97 ± 0.02	0.95 ± 0.01	0.92 ± 0.03
Business	**0.47 ± 0.01**	0.48 ± 0.01	0.47 ± 0.01	0.47 ± 0.01	0.48 ± 0.01	0.47 ± 0.01	0.48 ± 0.01	0.48 ± 0.01	0.47 ± 0.01
Education	**0.88 ± 0.03**	0.91 ± 0.02	0.94 ± 0.02	0.95 ± 0.03	0.95 ± 0.01	0.9 ± 0.02	0.95 ± 0.01	0.93 ± 0.01	0.9 ± 0.02
Entertain	**0.82 ± 0.04**	0.87 ± 0.04	0.9 ± 0.03	0.93 ± 0.03	0.91 ± 0.01	0.85 ± 0.03	0.94 ± 0.01	0.9 ± 0.01	0.86 ± 0.03
Health	**0.66 ± 0.04**	0.73 ± 0.06	0.67 ± 0.01	0.8 ± 0.09	0.77 ± 0.05	0.71 ± 0.05	0.77 ± 0.05	0.74 ± 0.04	0.71 ± 0.05
Recreation	**0.86 ± 0.02**	0.89 ± 0.02	0.89 ± 0.02	0.88 ± 0.03	0.92 ± 0.01	0.87 ± 0.02	0.97 ± 0.01	0.95 ± 0.01	0.88 ± 0.02
Reference	0.74 ± 0.08	0.74 ± 0.08	**0.71 ± 0.13**	0.78 ± 0.07	0.8 ± 0.04	0.76 ± 0.06	0.81 ± 0.05	0.83 ± 0.04	0.76 ± 0.06
Science	**0.91 ± 0.02**	0.94 ± 0.01	0.96 ± 0.01	0.93 ± 0.03	0.97 ± 0.01	0.94 ± 0.01	0.98 ± 0.01	0.95 ± 0.01	0.94 ± 0.01
Social	**0.70 ± 0.05**	0.72 ± 0.05	0.76 ± 0.13	0.88 ± 0.09	0.73 ± 0.09	0.72 ± 0.05	0.78 ± 0.07	0.74 ± 0.04	0.72 ± 0.05
Average	**0.74**	0.79	0.80	0.82	0.80	0.77	0.82	0.83	0.77

**Table 7:** Experimental results of multi-label feature selection methods in terms of Macro-F1 (mean ± std) using the Support Vector Machine (SVM) classifier.

Data set	MCMFS	PPT + MI	PPT + CHI	MIFS	D2F	MDMR	PMU	SCLS	LRFS
medical	**0.35 ± 0.06**	0.25 ± 0.05	0.26 ± 0.04	0.22 ± 0.05	0.19 ± 0.05	0.32 ± 0.07	0.19 ± 0.06	0.08 ± 0.01	0.32 ± 0.07
scene	**0.48 ± 0.09**	0.22 ± 0.09	0.21 ± 0.1	0.21 ± 0.15	0.46 ± 0.08	0.43 ± 0.07	0.47 ± 0.09	0.26 ± 0.05	0.44 ± 0.08
enron	0.12 ± 0.03	0.1 ± 0.03	0.07 ± 0.02	0.07 ± 0.02	**0.13 ± 0.04**	0.11 ± 0.03	0.13 ± 0.05	0.12 ± 0.03	0.1 ± 0.03
Arts	**0.09 ± 0.03**	0.06 ± 0.02	0.07 ± 0.02	0.07 ± 0.02	0.03 ± 0.00	0.08 ± 0.03	0.01 ± 0.01	0.03 ± 0.00	0.07 ± 0.02
Business	**0.05 ± 0.00**	0.05 ± 0.00	0.05 ± 0.00	0.04 ± 0.00	0.05 ± 0.00	0.05 ± 0.00	0.03 ± 0.00	0.04 ± 0.00	0.05 ± 0.00
Education	**0.07 ± 0.01**	0.06 ± 0.01	0.05 ± 0.01	0.03 ± 0.02	0.05 ± 0.01	0.06 ± 0.01	0.03 ± 0.01	0.04 ± 0.01	0.06 ± 0.01
Entertain	**0.13 ± 0.03**	0.11 ± 0.03	0.09 ± 0.02	0.06 ± 0.02	0.08 ± 0.01	0.12 ± 0.02	0.05 ± 0.00	0.07 ± 0.01	0.12 ± 0.02
Health	**0.15 ± 0.03**	0.13 ± 0.03	0.14 ± 0.03	0.06 ± 0.03	0.09 ± 0.01	0.14 ± 0.03	0.08 ± 0.01	0.09 ± 0.01	0.14 ± 0.03
Recreation	**0.11 ± 0.02**	0.1 ± 0.02	0.1 ± 0.02	0.09 ± 0.03	0.08 ± 0.01	0.11 ± 0.02	0.03 ± 0.00	0.04 ± 0.00	0.11 ± 0.02
Reference	**0.07 ± 0.01**	0.07 ± 0.01	0.07 ± 0.02	0.06 ± 0.02	0.04 ± 0.00	0.07 ± 0.01	0.03 ± 0.01	0.02 ± 0.00	0.07 ± 0.01
Science	**0.07 ± 0.02**	0.05 ± 0.02	0.05 ± 0.01	0.04 ± 0.02	0.02 ± 0.00	0.05 ± 0.02	0.01 ± 0.01	0.02 ± 0.00	0.05 ± 0.02
Social	**0.11 ± 0.03**	0.09 ± 0.02	0.09 ± 0.02	0.05 ± 0.03	0.07 ± 0.01	0.1 ± 0.03	0.05 ± 0.01	0.05 ± 0.01	0.1 ± 0.03
Average	**0.15**	0.11	0.10	0.09	0.11	0.14	0.09	0.07	0.14

**Table 8:** Experimental results of multi-label feature selection methods in terms of Macro-F1 (mean ± std) using the 3-Nearest Neighbors (3NN) classifier.

Data set	MCMFS	PPT + MI	PPT + CHI	MIFS	D2F	MDMR	PMU	SCLS	LRFS
medical	**0.25 ± 0.04**	0.16 ± 0.03	0.19 ± 0.02	0.16 ± 0.02	0.12 ± 0.02	0.19 ± 0.03	0.11 ± 0.02	0.06 ± 0.01	0.19 ± 0.03
scene	**0.54 ± 0.07**	0.37 ± 0.08	0.36 ± 0.08	0.29 ± 0.14	0.49 ± 0.05	0.51 ± 0.06	0.49 ± 0.07	0.37 ± 0.03	0.53 ± 0.07
enron	**0.13 ± 0.02**	0.12 ± 0.02	0.07 ± 0.01	0.09 ± 0.01	0.12 ± 0.01	0.12 ± 0.02	0.12 ± 0.02	0.11 ± 0.01	0.11 ± 0.02
Arts	**0.11 ± 0.03**	0.08 ± 0.02	0.1 ± 0.03	0.08 ± 0.03	0.06 ± 0.01	0.1 ± 0.02	0.06 ± 0.01	0.07 ± 0.02	0.1 ± 0.02
Business	**0.1 ± 0.01**	0.08 ± 0.01	0.09 ± 0.01	0.09 ± 0.02	0.07 ± 0.01	0.09 ± 0.01	0.05 ± 0.01	0.07 ± 0.01	0.08 ± 0.01
Education	**0.09 ± 0.02**	0.08 ± 0.02	0.08 ± 0.02	0.04 ± 0.02	0.06 ± 0.01	0.07 ± 0.01	0.06 ± 0.01	0.06 ± 0.01	0.07 ± 0.01
Entertain	**0.14 ± 0.03**	0.13 ± 0.02	0.11 ± 0.02	0.08 ± 0.02	0.11 ± 0.01	0.13 ± 0.02	0.08 ± 0.01	0.09 ± 0.01	0.14 ± 0.02
Health	**0.14 ± 0.03**	0.11 ± 0.02	0.12 ± 0.03	0.05 ± 0.03	0.09 ± 0.01	0.12 ± 0.02	0.09 ± 0.01	0.09 ± 0.01	0.12 ± 0.02
Recreation	**0.13 ± 0.02**	0.1 ± 0.01	0.11 ± 0.02	0.12 ± 0.03	0.08 ± 0.01	0.12 ± 0.02	0.05 ± 0.01	0.07 ± 0.01	0.12 ± 0.02
Reference	**0.08 ± 0.01**	0.07 ± 0.01	0.08 ± 0.02	0.07 ± 0.01	0.04 ± 0	0.07 ± 0.01	0.03 ± 0.01	0.04 ± 0.01	0.07 ± 0.01
Science	**0.08 ± 0.02**	0.05 ± 0.01	0.07 ± 0.01	0.06 ± 0.02	0.04 ± 0.01	0.07 ± 0.02	0.03 ± 0.01	0.03 ± 0	0.06 ± 0.01
Social	**0.12 ± 0.02**	0.08 ± 0.01	0.1 ± 0.01	0.07 ± 0.03	0.06 ± 0.01	0.09 ± 0.01	0.05 ± 0.01	0.05 ± 0	0.09 ± 0.01
Average	**0.16**	0.12	0.12	0.10	0.11	0.14	0.10	0.09	0.14

**Table 9:** Experimental results of multi-label feature selection methods in terms of Micro-F1 (mean ± std) using the SVM classifier.

Data set	MCMFS	PPT + MI	PPT + CHI	MIFS	D2F	MDMR	PMU	SCLS	LRFS
medical	**0.79 ± 0.05**	0.73 ± 0.05	0.74 ± 0.07	0.71 ± 0.11	0.63 ± 0.07	0.76 ± 0.05	0.63 ± 0.08	0.37 ± 0.01	0.76 ± 0.05
scene	**0.50 ± 0.08**	0.25 ± 0.1	0.24 ± 0.11	0.24 ± 0.16	0.48 ± 0.07	0.46 ± 0.07	0.49 ± 0.08	0.3 ± 0.05	0.47 ± 0.07
enron	**0.51 ± 0.03**	0.47 ± 0.04	0.35 ± 0.02	0.37 ± 0.03	0.51 ± 0.03	0.47 ± 0.05	0.5 ± 0.04	0.49 ± 0.03	0.45 ± 0.06
Arts	**0.18 ± 0.05**	0.14 ± 0.05	0.12 ± 0.04	0.17 ± 0.05	0.08 ± 0.01	0.17 ± 0.05	0.03 ± 0.02	0.07 ± 0.02	0.16 ± 0.05
Business	**0.68 ± 0.00**	0.68 ± 0.00	0.68 ± 0.00	0.67 ± 0.00	0.67 ± 0.00	0.68 ± 0.00	0.67 ± 0.00	0.67 ± 0	0.68 ± 0.00
Education	**0.23 ± 0.05**	0.2 ± 0.04	0.13 ± 0.04	0.12 ± 0.06	0.12 ± 0.02	0.21 ± 0.05	0.08 ± 0.01	0.14 ± 0.02	0.21 ± 0.04
Entertain	**0.27 ± 0.06**	0.23 ± 0.06	0.17 ± 0.05	0.11 ± 0.05	0.16 ± 0.01	0.26 ± 0.06	0.1 ± 0.01	0.15 ± 0.02	0.25 ± 0.06
Health	**0.50 ± 0.02**	0.45 ± 0.07	0.47 ± 0.03	0.39 ± 0.05	0.42 ± 0.01	0.47 ± 0.04	0.39 ± 0.03	0.41 ± 0	0.48 ± 0.03
Recreation	**0.20 ± 0.04**	0.19 ± 0.03	0.17 ± 0.04	0.18 ± 0.05	0.14 ± 0.02	0.2 ± 0.04	0.04 ± 0	0.07 ± 0.01	0.2 ± 0.04
Reference	0.32 ± 0.04	0.35 ± 0.07	**0.35 ± 0.14**	0.33 ± 0.1	0.31 ± 0.04	0.34 ± 0.06	0.27 ± 0.05	0.26 ± 0.04	0.34 ± 0.06
Science	**0.15 ± 0.04**	0.12 ± 0.03	0.09 ± 0.03	0.11 ± 0.05	0.05 ± 0.01	0.13 ± 0.03	0.02 ± 0.02	0.06 ± 0.01	0.13 ± 0.03
Social	**0.45 ± 0.08**	0.42 ± 0.07	0.38 ± 0.14	0.2 ± 0.12	0.4 ± 0.07	0.43 ± 0.07	0.31 ± 0.07	0.38 ± 0.05	0.43 ± 0.07
Average	**0.40**	0.35	0.33	0.30	0.33	0.38	0.29	0.28	0.38

**Table 10:** Experimental results of multi-label feature selection methods in terms of Micro-F1 (mean ± std) using the 3NN classifier.

Data set	MCMFS	PPT + MI	PPT + CHI	MIFS	D2F	MDMR	PMU	SCLS	LRFS
medical	**0.69 ± 0.04**	0.62 ± 0.04	0.64 ± 0.06	0.61 ± 0.1	0.53 ± 0.04	0.64 ± 0.03	0.52 ± 0.04	0.35 ± 0.01	0.64 ± 0.03
scene	**0.55 ± 0.06**	0.39 ± 0.06	0.38 ± 0.06	0.34 ± 0.11	0.49 ± 0.04	0.52 ± 0.05	0.5 ± 0.05	0.38 ± 0.02	0.54 ± 0.05
enron	**0.49 ± 0.02**	0.45 ± 0.01	0.34 ± 0.03	0.41 ± 0.02	0.47 ± 0.03	0.44 ± 0.04	0.47 ± 0.02	0.44 ± 0.03	0.42 ± 0.05
Arts	**0.26 ± 0.05**	0.17 ± 0.05	0.24 ± 0.04	0.18 ± 0.05	0.15 ± 0.03	0.25 ± 0.04	0.14 ± 0.03	0.17 ± 0.03	0.25 ± 0.04
Business	**0.67 ± 0.01**	0.67 ± 0.00	0.66 ± 0.01	0.65 ± 0.08	0.66 ± 0.00	0.67 ± 0.01	0.65 ± 0.04	0.60 ± 0.12	0.67 ± 0.01
Education	**0.26 ± 0.03**	0.24 ± 0.04	0.28 ± 0.04	0.16 ± 0.06	0.19 ± 0.03	0.23 ± 0.03	0.18 ± 0.04	0.19 ± 0.03	0.23 ± 0.03
Entertain	0.27 ± 0.05	**0.28 ± 0.05**	0.21 ± 0.05	0.22 ± 0.08	0.24 ± 0.03	0.26 ± 0.04	0.22 ± 0.05	0.22 ± 0.03	0.27 ± 0.03
Health	0.37 ± 0.09	**0.38 ± 0.07**	0.37 ± 0.14	0.2 ± 0.07	0.37 ± 0.05	0.38 ± 0.06	0.36 ± 0.04	0.37 ± 0.06	0.38 ± 0.05
Recreation	**0.25 ± 0.03**	0.19 ± 0.02	0.21 ± 0.04	0.23 ± 0.05	0.16 ± 0.02	0.23 ± 0.04	0.09 ± 0.02	0.12 ± 0.02	0.23 ± 0.03
Reference	**0.46 ± 0.04**	0.41 ± 0.05	0.39 ± 0.13	0.35 ± 0.09	0.36 ± 0.05	0.43 ± 0.05	0.35 ± 0.04	0.29 ± 0.05	0.43 ± 0.04
Science	**0.18 ± 0.04**	0.17 ± 0.03	0.12 ± 0.03	0.17 ± 0.02	0.12 ± 0.02	0.16 ± 0.03	0.1 ± 0.02	0.15 ± 0.03	0.16 ± 0.03
Social	**0.46 ± 0.05**	0.4 ± 0.06	0.44 ± 0.1	0.39 ± 0.05	0.39 ± 0.05	0.42 ± 0.06	0.36 ± 0.05	0.37 ± 0.04	0.41 ± 0.05
Average	**0.41**	0.36	0.36	0.33	0.34	0.38	0.33	0.30	0.38

**Table 11:** Running time (seconds).

Data Set	MCMFS	D2F	MDMR	PMU	SCLS	LRFS	PPT + MI	PPT + CHI	MIFS
medical	142.2	10,698.5	9910.0	11521.6	38.2	6961.9	1.2	8.6	30.3
scene	23.5	244.1	246.1	257.0	5.5	147.0	0.4	1.8	47.4
enron	267.3	22,164.0	20,583.0	25749.6	93.6	16,631.9	0.8	6.4	41.2
Arts	111.9	4355.8	4185.4	5002.2	39.6	3421.2	1.0	4.1	35.5
Business	107.4	4536.1	4361.8	5354.0	41.5	4079.8	1.0	3.1	41.9
Education	161.2	8397.3	7700.0	9037.2	59.1	6323.1	1.2	4.3	99.3
Entertain	194.4	6809.9	6539.2	7386.6	54.3	4519.1	1.4	5.0	98.7
Health	195.1	9376.0	9086.8	10,797.1	68.4	7523.4	1.3	4.8	17.2
Recreation	183.4	6394.2	6127.6	7016.8	51.7	4369.0	1.3	4.6	39.5
Reference	313.5	16,269.8	15,637.3	18,483.6	96.2	11,685.1	1.8	5.7	57.5
Science	291.4	17,419.9	16,665.2	20,137.1	102.7	14,344.9	1.6	6.6	60.0
Social	553.8	34,108.8	33,195.6	37,246.0	156.1	23,754.5	2.8	9.0	43.4
